# The Use of the Microscope in the Differential Diagnosis of Morbid Growths

**Published:** 1871-07

**Authors:** S. Fleet Speir

**Affiliations:** Surgeon to the Brooklyn City Hospital; Surgeon to the Brooklyn Eye and Ear Infirmary; Surgeon to the Tumor and Cancer Department of the Brooklyn Dispensary; Fellow of the New York Academy of Medicine; Member of the New York Pathological Society, &c.


### ARTICLE VI.

### *The Use of the Microscope in the Differential Diagnosis of Morbid Growths,*

WITH A NEW METHOD FOR DETERMINING THE DIAGNOSIS, PROGNOSIS AND TREATMENT OF TUMORS AND CANCERS.

By S. Fleet Speir, M. D.

Surgeon to the Brooklyn City Hospital; Surgeon to the Brooklyn Eye and Ear Infirmary; Surgeon to the Tumor and Cancer Department of the Brooklyn Dispensary; Fellow of the New York Academy of Medicine; Member of the New York Pathological Society, &c.

READ BEFORE THE NEW YORK ACADEMY OF MEDICINE, APRIL 6TH, 1871.

We find, then, for our present purpose, that morbid growths may be divided into three classes, according as they are more or less susceptible to the influence of treatment, as follows:

1st. Growths which are curable by the application of local and constitutional remedies, without operation.

2d. Growths which are curable by operation, followed by constitutional and local after-treatment.

3d. Growths which are incurable, or which resist all the known means of cure, and are necessarily fatal.

If, now, this division is correct, and we can find means of determining to which class a given growth belongs, the mode of treatment for that particular case may be at once laid down with great accuracy.

It was claimed, in the chapter on diagnosis, that the microscope, applied to the examination of tumors according to the rules there laid down, was capable of itself, without the aid of the history of the symptoms of the case, to decide to which of the three classes there mentioned (viz: 1st. Malignant. 2d. Semi-Malignant. 3d. Benign) a given tumor belonged.

It is now claimed that the microscope is equally capable, according to the same rules, prescribing appropriate rules of treatment according to the quality of a given tumor.

However, in searching for a solution of the question of treatment applicable to a given case, we must lay aside the first classification of morbid growths according to their malignity, and substitute the classification ; as here proposed, according to their curability and then we have, instead of the Malignant, Semi-Malignant, and Benign, the following divisions:

1st. Tumors which are curable by treatment, without operation.

2d. Tumors which are curable by operation, and after-treatment.

3d. Tumors which are incurable.

The reason for this change in classification for the purpose of deciding an appropriate treatment will be apparent, when we refer to the chapter of diagnosis, where it will be seen that the classification, according to the malignity of tumors, while it served well its purpose of general diagnosis, could not afford the needed data for a special basis of treatment ; for there, it will be seen, that a semi-malignant growth, for example, may have a certain range of malignity, as shown in the four divisions of which it is capable ; and vary, from the mildest form approaching the benign, to the more severe forms, and become in the end a purely malignant growth. Therefore, we must have a nicer classification, and one which will bear an examination with special reference to treatment. That furnished by the relative curability of tumors seems to me to answer our purpose best, for the reason that it has a bearing upon the subdivisions of the classification according to malignity; and it is, in fact, necessary in order to make the doctrine of heterology and homology, as here set forth, applicable in determining a special treatment for each case.

Adopting this, then, as our classification for the present, we find that it affords us an easy means of arriving at the treatment most applicable to any of the forms of morbid growths, in the following manner:

We have, first, to make the diagnosis as to the variety of the growth, and class it as the encephaloid, scirrhus, or, other variety, according to its structure, as directed in the chapter on diagnosis. This will have settled the point next in order, viz.: the relation of the cells of the tumor to the other tissues and to its own cells, as regards their homology or heterology. And, now, adopting the conclusions drawn in the first part of the paper, and applying them to the question of the curability of morbid growths, we find that they arrange themselves as follows, according to their cellular relations to the other tissues, and to their own cells:—

A tumor which is found, upon microscopic examination, to be

| | |
|---|---|
| Heterologous as to the other tissue and homologous as to its own cells | *Is incurable.* |
| A tumor which is<br>Homologous as to the other tissues, and homologous as to its own cells;<br>or which is<br>Heterologous as to the other tissues, and heterologous as to it sown cells, | *Is curable by operations, and after-treatment.* |
| A tumor which is<br>Homologous as to the other tissues and heterologous as to its own cells, | *is curable by local and constitutional treatment or by operation.* |

This table will be found correct as a rule, but it must be remembered that all growths, with one exception (the encephaloid), may have a tendency to progress either in one direction or the other. For example, a semi-malignant growth may have a tendency to become purely malignant; or it may, on the contrary, tend to become benign. And this must always be covered, at the time of the microscopic examination, and is to be recognized by the relation of the cells of the growth to its own cells.

If the specimen under examination contains cells which, in the main, are homologous as their own cells, and yet in some places they may be found to show a slight tendency to become heterologous as to their own cells; it may be taken for granted that the growth is to a certain extent curable, and the degree of curability of a tumor will be in direct proportion to the extent of this tendency of the cells of the growth to become heterologous as to their own cells; and the possibility of inducing them to become such by treatment.

Thus, a tumor may be pronounced curable, although it may belong to the semi-malignant group; and we may pick out, so to speak, from the malignant or semi-malignant class, such as are manifesting a greater or less tendency of this kind, and place them among the curable growths.

It is in this way, also, that we are to decide whether operation or local and constitutional treatment will be appropriate for the case; or whether the patient will live longer by leaving the case to nature, the disease being incurable, and interference dangerous.

Again, a tumor which has presented the characters of curability in the relations of its cell structure for a length of time, may suddenly take on a homologous tendency; and in proportion as it does this, it would be classed as incurable by local or constitutional treatment, and would necessitate immediate operation and local after-treatment.

The table of comparison here presented will perhaps make these points more clear, and show the advantage of thus classifying growths according to their curability, for the purpose of defining the general treatment.

### TABLE OF COMPARISON.

Classification according to the degree of malignity for purposes of diagnosis.

| | |
|---|---|
| Heterologous as to the other tissues and Homologous as to its own cells or Homologous as to the other tissues, and Homologous as to its own cells. | *Malignant.* |
| Heterologous as to the other tissues, and Heterologous as to its own cells. | *Semi-Malignant.* |
| Homologous as to the other tissues, and Heterologous as to its own cells. | *Benign.* |

Classifications according to the degree of curability for determining the treatment.

| | |
|---|---|
| Heterologous as to the other tissues, and Homologous as to its own cells. | *Incurable.* |
| Homologous as to the other tissues, and Homologous as to its own cells; or Heterologous as to the other tissues, and Heterologous as to its own cells. | *Curable by operation and constitutional and local after treatment.* |
| Homologous as to the other tissues, and Heterologous as to its own cells. | *Curable by constitutional operation and local treatment without operation.* |

It is to be understood that the specimen is always examined first as to the other tissues, and second as to its own cells; always putting the relation of the cells to the other tissues first. Thus, for example, selecting the malignant form we would call it a heterologous-homologous growth, affording by the use of a single expression, a means of implying the degree of malignity, the curability, and the cellular arrangement of the growth—as homologous-homologous, or heterologous-homologous, etc.

Thus far we have only determined upon the curability of tumors, and the kind of treatment or operation which is indicated by the nature of the profit as determined by the microscope in the examination of the cellular elements. We must now leave the microscope as an aid, and come to the part concerned in the application of the treatment, a knowledge of which can only be derived from clinical experience and observation; and I must confess that many, and perhaps most, of my readers may have had more of both these than myself; and, therefore, I touch upon them with more hesitation. Nevertheless, it is due to the method which has been thus far followed up boldly to continue on, even at the risk of offending some who may have had more experience than myself and think differently. And yet, when we come to the substance of the matter, we find that the question of a specific treatment, or anything like a specific treatment, involves a great deal more of what we do not know, and what we cannot do, than it does of plain, successful treatment. So much has been recommended, and so many specific methods for the treatment of cancer have been tried in vain, that it would be a useless task for any one to endeavor to determine which is the good and which is the useless among them.

I prefer, therefore, to "stick to my text," and instead of presenting a review of the various substances which have been recommended for the cure of cancer, to offer such a method and such means of treatment as have seemed to be indicated upon a careful histological study of the nature of growth and decay of tumors, as well as the manner in which they sometimes undergo spontaneous resolution and cure; and to suggest such methods of cure as promise most—from such a study—to be successful.

In studying nature's methods for the removal of morbid growths, we find that she has two:

1st. *That by spontaneous resolution.*

This is accomplished by reversing the order of things which produced the growth in the first instance, namely: the conversion of the abnormal cellular development into a normal cell growth; and also, but more rarely, by fatty degeneration, and absorption of the abnormal cellular parts, and a subsequent production of normal cells, with a heterologous combination.

2d. *That by sloughing.*

This method rarely accomplishes the cure of a tumor, for the reason that nature usually finds herself unable to complete the process; for the slough rarely extends beyond the diseased parts, and there is, therefore, always left a starting point for the reproduction of the growth.

We have here two valuable lessons by which the profession have profited more or less in adopting them as so many means of treatment. The first method is included in our local treatment by the application of resolvents. The second method we imitate in the application of caustics, and in operations for the removal of tumors.

To these there may be added a third method, not used by nature; and we shall have completed the list of our resources for treatment, namely: a constitutional treatment, intended to aid in the cure by placing the patient in the most favorable conditions for recovery, or by adding to the blood elements which are antagonistic to the growth.

We have, therefore, three modes of procedure:

1st. *Operation for Removal.*

2d. *External or Local Treatment.*

3d. *Internal or Constitutional Treatment.*

It must be acknowledged that this is quite a formidable array; and if the same amount of curative force could be brought to bear upon most other diseases which we have to combat, there would be few among them that could resist it.

Even among the growths which we have been studying, we find but one class which is positively and necessarily incurable, viz: the heterologous-homologous form. The others are all more or less susceptible of cure, through the application of one or the other, or a combination of these methods, when they are applied faithfully and persistently: provided, however, the methods of treatment employed are adapted to the case. The method to be used must be selected especially for the case. The rules laid down above are to be used for this purpose.

In a general way, it may be said that the *malignant forms* require previous constitutional treatment, operation, and then a constitutional and after-treatment.

The *semi-malignant* forms require in general the same treatment as the malignant; but in those cases where the tendency is towards the higher grades of this form, operation is unnecessary.

Benign growths may be treated by operation, if desirable; but they are also amenable to a local treatment.

Under the head of operation for removal is included all operations for the removal of the whole or a large part of a growth "*en masse*," whether it be by caustic, injection, the ligature or the knife.

External or local treatment includes all the applications which are used for the resolution of tumors on account of the power they possess of stimulating or retarding cell-growth, or increasing absorption, when applied outwardly.

Internal or constitutional treatment includes those remedies which, by their action upon the blood and stomach, favor the normal or healthy action of the organs of the body, and thereby tend to present what is known as cachexia.

It is no part of the object of this paper to offer a plan of specific treatment. Indeed, it is quite opposed to it. Nevertheless, it will be desirable to give an example of the author's method of carrying out the details of these general principles of treatment.

1st. Rest, in all cases. Free the part in which the growth is situated from all sources of irritation.

2d. For tumors covered by the integument, apply a liniment composed of potass., iod. and belladona; and give appropriate constitutional treatment.

3d. For epithelioma and all open cancers exposing a granulating surface, apply strong caustics and give appropriate constitutional treatment; and operation, should these fail.

Encephaloid must be extirpated at once. This is the only treatment for this form; and if there is any hope, it will be realized only by a persistent use of local and constitutional remedies after the removal.

Scirrhus should be extirpated, and followed by local and constitutional after-treatment.

Benign tumors may be extirpated or treated locally as the case may seem to reqiure.

It is important in some cases to see that the patient is in a proper condition, and to give such a course of constitutional treatment before the operation as the case may demand.

We occasionally find tumors which have a benign appearance and character until they are disturbed by operation, when they recur and take on a malignant course, and become rapidly fatal. These tumors can be recognized by the microscope, and by it they are shown to be of the semi-malignant type. When we have been able to determine that the growth is of such a character, it may better in some cases when it has not yet taken on its malignant course, to treat it locally and constitutionally, and defer operative interference, which seems to be sometimes the starting point of a more rapid growth; but if, on account of its location, or other reason, is is desirable to operate, a previous course of local and constitutional treatment should be used, in order to put the tissues in as favorable condition as possible for recovery.

I has seemed to me that a combination of iodide of potassium with belladonna, as a liniment, has a powerful effect upon the cellular growth of tissues ; and I have been enabled by the persistent use of it, to dissipate growths which at first appeared only capable of removal by operation. I, therefore, mention this as an example of local applications in cases where the tumor is covered by integument.

Caustic applications are often sufficient to cure the smaller growths, and may even be successful in large cancers. It may often be preferred on the ground of dander in the use of the knife, by reason of the number or size of the blood vessels, etc., and also on the ground that it may be made to accomplish the effects of operation and local after treatment at the same time. The most useful caustics are the preparations of zinc, arsenic, acid nitrate of mercury, etc

As to constitutional treatment, each case must furnish its own indications. These indications are various, and may sometimes be fulfilled by one remedy and sometimes by another.

The following are some of the remedies which promise to be useful on account of their influence upon cell growth, and upon the blood. Iodide of potassium, chlorate of potash, cod liver oil, iron, belladonna, arsenic phosphate of ammonia, etc.

The iodide of potassium with phosphate of ammonia, used freely, is certainly of great value in some cases of morbid growths, and especially in conjunction with the liniment above recommended.

Such are the impressions which I have received of the treatment of morbid growths. Such are the remedies upon which we must rely, upon the absence of the specific which has been sought after thus far in vain. Should a specific be found, doubtless its strength will consist in its power to control and direct cell development and growth.

Let us hope that by a diligent search such a remedy will soon be found ; and, meanwhile, let us apply faithfully the means which are even now at our disposal, and which are in many cases all that is needed to effect a cure.—*Medical Gazette.*

